# The Role of Artificial Intelligence in Obesity Risk Prediction and Management: Approaches, Insights, and Recommendations

**DOI:** 10.3390/medicina61020358

**Published:** 2025-02-19

**Authors:** Lillian Huang, Ellen N. Huhulea, Elizabeth Abraham, Raphael Bienenstock, Esewi Aifuwa, Rahim Hirani, Atara Schulhof, Raj K. Tiwari, Mill Etienne

**Affiliations:** 1 School of Medicine, New York Medical College, 40 Sunshine Cottage Road, Valhalla, NY 10595, USArhirani2@student.nymc.edu (R.H.);; 2 Graduate School of Biomedical Sciences, New York Medical College, Valhalla, NY 10595, USA; 3 Department of Neurology, New York Medical College, Valhalla, NY 10595, USA

**Keywords:** obesity, artificial intelligence, machine learning, childhood obesity

## Abstract

Greater than 650 million individuals worldwide are categorized as obese, which is associated with significant health, economic, and social challenges. Given its overlap with leading comorbidities such as heart disease, innovative solutions are necessary to improve risk prediction and management strategies. In recent years, artificial intelligence (AI) and machine learning (ML) have emerged as powerful tools in healthcare, offering novel approaches to chronic disease prevention. This narrative review explores the role of AI/ML in obesity risk prediction and management, with a special focus on childhood obesity. We begin by examining the multifactorial nature of obesity, including genetic, behavioral, and environmental factors, and the limitations of traditional approaches to predict and treat morbidity associated obesity. Next, we analyze AI/ML techniques commonly used to predict obesity risk, particularly in minimizing childhood obesity risk. We shift to the application of AI/ML in obesity management, comparing perspectives from healthcare providers versus patients. From the provider’s perspective, AI/ML tools offer real-time data from electronic medical records, wearables, and health apps to stratify patient risk, customize treatment plans, and enhance clinical decision making. From the patient’s perspective, AI/ML-driven interventions offer personalized coaching and improve long-term engagement in health management. Finally, we address key limitations and challenges, such as the role of social determinants of health, in embracing the role of AI/ML in obesity management, while offering our recommendations based on our literature review.

## 1. Introduction

Obesity has tripled in global prevalence since 1975, and it is predicted that in 2025, 177 million people will be morbidly obese, 1 billion people would be obese, and 2.7 billion adults would be overweight [1,2]. Obesity has been noted as a risk factor for numerous diseases, such as multiple types of cancers, obstructive sleep apnea, cardiovascular disease, and more [1]. Childhood obesity has also been linked with poor psychological and emotional health including increased stress, depressive symptoms, and low self-esteem [3]. Healthcare costs are high due to the many comorbidities linked to childhood obesity. The World Health Organization has projected that conditions related to one’s lifestyle, including those related to obesity, will account for 30% of global deaths by 2030, highlighting the importance of obesity prevention [4].

Artificial intelligence (AI) is a field in computer science that utilizes algorithms to create systems that are capable of performing tasks that typically require human intelligence, such as decision-making, visual recognition, language processing, and more [5,6]. AI has become important in the healthcare field, especially regarding chronic patient management and disease prevention. AI can improve patient satisfaction through more informative and efficient care and easier patient follow-up. Since the COVID-19 pandemic, there has been a rapid increase in AI in healthcare such as telemedicine and chatbots [7]. Machine learning (ML), another development of computer systems, has been utilized in analyzing datasets without explicit programming [8]. While AI is an umbrella term mainly used to describe computer programs that have the capability to predict outcomes like humans, ML specifically functions with an algorithm and an abundance of data without being explicitly programmed to do so. ML utilizes either supervised learning, where data are labeled beforehand and input is tagged with preferred outcomes, or unsupervised learning, where ML functions to generate recurring patterns in data without labels. ML also has the capability to create a supervised learning model by itself, termed ‘self-supervised learning’ [6]. For example, ML has been used in disease prediction and detection but has varying levels of accuracy based on feature set, training dataset, algorithm, and more [9]. Deep learning is a subset of ML that employs neural networks with multiple layers to extract patterns from large datasets and has already been applicable in image and speech recognition within multidimensional data [6]. Deep learning itself has assessment algorithms and predicting labels that have developed several outcome varieties that build upon ML, such as prognosis, immune score, histological subtyping, microsatellite instability, and more [10]. Furthermore, deep learning has aided in regulating and monitoring multiple brain imaging modalities such as positron emission tomography (PET) scans, computed tomography (CT) scans, electroencephalograms, convolutional neural networks (CNN), recurrent neural networks (RNN), and generative adversarial networks (GAN) [10].

**Figure 1:**
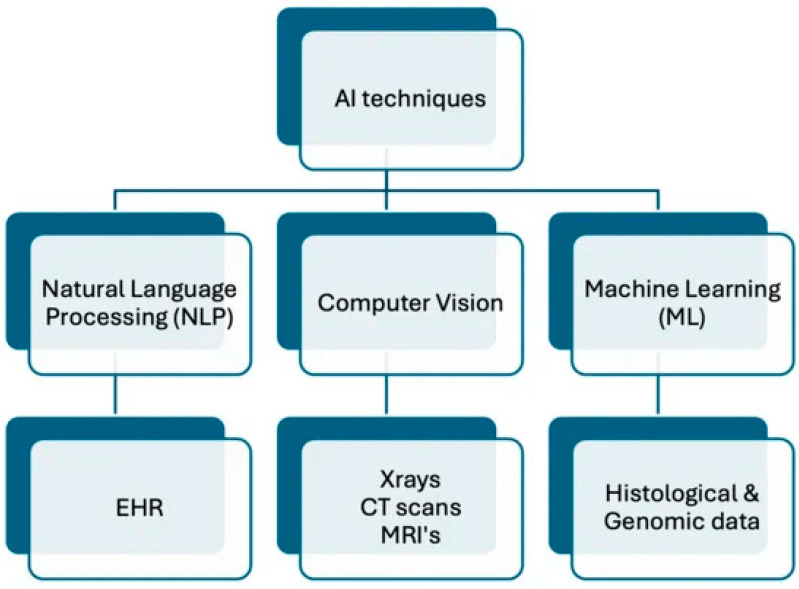
Hierarchical relationships between AI, ML, reinforcement learning, and natural language processing—key techniques focused in this review for potential in obesity care, adapted from Hirani et al. [7].

CNNs are extremely helpful in neuroscience by predicting pathology and disease progressions through the identification of brain structures and the analysis of MRI, CT scans, and physiological data [10]. Specifically, speech signal data, unlike CNNs, provide the benefit of analyzing and predicting changes in EEGs and MEGs [10]. In obesity research, RNNs can be employed to predict obesity-related outcomes by analyzing longitudinal health data that present in a sequential structure or even uncover regulatory networks among genes proteins that contribute to obesity. To facilitate the continued advancements of these AI models, feedback and coding improvements are vital. A diagram summarizing the overlapping relationships of aforementioned AI techniques is illustrated in Figure 1.

Given the rapidly expanding role of AI in obesity management, this study aims to provide a narrative review of current AI applications in obesity risk prediction, prevention, and treatment. A literature search was conducted using PubMed, Google Scholar, SpringerLink, and MDPI databases, focusing on relevant studies published in the fields of AI, machine learning, and obesity care. Keywords included “artificial intelligence”, “machine learning”, “obesity”, “childhood obesity”, “interventions”, “overweight”, “BMI”, “coaching”, “precision nutrition”, and “gamification”. This review synthesizes existing research, highlighting AI-driven innovations in obesity management while discussing clinical implications, potential benefits, and challenges associated with implementing these emerging technologies.

### 1.1. AI/ML in Healthcare

AI and ML have already proven useful in many clinical fields, like dermatology, radiology, cardiology, neurology, and pharmacology with visual recognition and analysis of longitudinal data. [11,12]. In the field of dermatology, AI research focuses on early skin cancer detection through dermoscopic images [13]. Over the last two decades, ML has aided in computer-aided diagnosis in radiology by integrating radiology, pathology, and genomics to aid in efficiency for work-flow and bring about an integrated diagnostic service [14,15]. Deep learning neural networks have improved radiologists performance in breast cancer screening as well; a recent study using over 200,000 breast screening exams for ML to gain accuracy resulted in the same diagnoses as experienced radiologists when presented with the same data [16]. Deep learning demonstrates its capacity to interpret electrocardiograms and echocardiograms and subsequently predict systemic phenotypes in echocardiograms that modify cardiovascular risk by analyzing flow volume and metrics of cardiac function [17].

### 1.2. AI/ML in Obesity Care

AI and ML have been effectively used across many areas of healthcare and hold equal promise in predicting and managing obesity through analyzing complex health data, identifying risk factors, and assisting physicians to holistically care for their patients with obesity. Through implementing AI/ML, patients receive personalized meal plans and a more streamlined process of follow-ups in their health [18]. Other AI and ML techniques used in obesity care are predictive modeling to interpret the risk of obesity [19], image analysis such as for dietary assessment [20], or obesity monitoring [21], AI chatbots [22], and wearables.

AI models such as Gradient Boosting, XGBoost, and Random Forest have demonstrated high accuracy in predicting obesity by analyzing physical descriptions, eating habits, and other health-related data [23]. Synthetic data generation is an ML model that creates artificial datasets, potentially offering a cost-effective alternative to time and labor spent collecting real-world data while addressing patient privacy concerns. This approach enables researchers to model and predict obesity-related pathologies and disease progression with minimal ethical and logistical constraints [24]. Additionally, human-in-the-loop is another example of an AI model that incorporates human expertise into decision-making or data generation workflows. This approach ensures the timely identification of biases and potentially harmful AI models while creating a positive feedback loop to enhance the ML process [25]. This is especially important for reducing stigmatization and negative internalization in the context of childhood obesity in particular, as this is a highly formative period for development of self-perception and body image. With this review’s focus on childhood obesity, AI models like the DeepHealthNet framework have been able to accurately predict 88.42% of adolescent obesity by analyzing factors such as height, weight, physical activity levels, and waist circumference [26].

### 1.3. AI in Childhood Obesity Care

As of 2020, 19.7% of children in the United States have been obese, a significant increase from 16% in 2002 and 11% in 1994 [27,28]. Childhood obesity prediction and management is particularly important given that it often precedes adult obesity and comorbid chronic diseases. By utilizing AI and ML to increase health outcomes in childhood and adolescence, healthy habits are instilled, which, according to the American Heart Association, may reduce the risk of adult obesity and heart disease, thereby improving quality of life and reducing obesity-related healthcare costs [29,30,31,32]. When leveraging AI to address childhood obesity, it is crucial to consider approaches that resonate with younger populations, such as gamifying mundane or disliked activities to encourage exercise and healthy eating habits. According to 2023 guidelines from the American Academy of Pediatrics (AAP), the “dose” threshold of 26 h of intensive behavioral intervention over a 3–12-month period can lead to a reduction in BMI. Additionally, the AAP calls for usage of multicomponent treatment modalities including physical activity and lifestyle changes, in order to maximize positive outcomes in childhood obesity treatment [3]. AI-powered tools, like health-focused apps with virtual coaching and “active video games” or “exergames”, integrate ML seamlessly into children’s daily lives, promoting healthier lifestyle choices in an engaging way—efficiently addressing the AAP guidelines for multifactorial treatment and promoting engagement to reach the 26-contact-hour threshold. For example, a systematic review and meta-analysis of 23 studies on game-based interventions demonstrated their effectiveness in improving nutritional knowledge, habits, and body composition, showing significant gains in nutrition-related understanding post-intervention [33]. Additionally, a narrative review highlighted the benefits of “exergames”—digital games requiring physical movement—which were found to enhance physical fitness, reduce body weight in overweight children, and improve VO_2_ max, among other health metrics [34]. As these strategies evolve, we recommend remaining attuned to what works best for children, considering individual preferences, family dynamics, cultural influences, and the importance of creating a supportive environment to maximize the impact of AI-driven solutions in fostering healthier futures.

Table 1 explores the role of AI/ML in the prediction and management of obesity, with a special focus on childhood obesity. With telehealth, mobile health applications (mHealth), and wearable devices to gather patient data, AI/ML has the potential to enhance accessibility and cost-efficacy of the treatment and prevention of obesity-related morbidity. In the process of drug discovery, ML can predict protein–protein interactions, structure, and drug dose drug delivery effectiveness, thereby resulting in new pharmacology passing phase II clinical trials in a time and cost-effective method [6]. With ML and deep learning, vast amounts of data can be processed from genetic information, electronic health records, and lifestyle factors, alongside other health metrics to predict obesity risk.

### 1.4. Healthcare Provider Perspectives on AI in Obesity Care

Healthcare providers are key players in supporting obesity management, yet they face significant challenges in helping patients make sustainable lifestyle changes in diet, exercise, and behavior. Obesity rates continue to rise [35], suggesting that current counseling approaches may not fully address the multifaceted challenges of long-term weight management. Overweight and obese individuals contribute to higher healthcare utilization [63]; while they engage with healthcare more frequently, their visits may not be effectively structured to support sustainable weight management.

Lifestyle modifications remain the most accessible intervention for the majority of patients. However, these approaches demand consistent motivation, resources, and sustained efforts, which many patients find difficult to maintain or have access to. Emerging data on anti-obesity medications and bariatric surgery outcomes [64] continue to shape treatment paradigms, but lifestyle interventions remain a cornerstone of care. Here, AI presents a unique opportunity to enhance traditional models by offering personalized, data-driven solutions to improve patient outcomes.

AI can bridge gaps in current practices by delivering deeper insights into patient behaviors and challenges while enabling healthcare providers to offer tailored, evidence-based interventions. This segment explores the healthcare provider perspective, focusing on how AI can enhance clinical practice and empower healthcare providers in obesity management.

### 1.5. mHealth Applications and Wearable Health Technology

mHealth applications and wearable technology (e.g., activity tracker wristbands or rings) have shown promise in personalizing obesity management, promoting weight reduction, increasing physical activity, and enhancing overall quality of life [65,66]. A meta-analysis of 12 studies evaluating mobile phone app interventions found that their use was associated with reductions in body weight (−1.07 kg) and body mass index (BMI) (−0.45 kg/m^2^) [65]. These findings highlight the potential of mobile technology to complement traditional obesity care approaches.

The Evident 3 trial—a randomized, multicenter clinical study—further illustrates the impact of mHealth interventions. This trial compared an intervention group (smartphone app, activity tracker wristband, and brief counseling) versus control group (counseling only) on weight loss and improving body composition, physical activity, and caloric intake in adults categorized as overweight or obese [67]. At the 3-month mark, the intervention group experienced an average weight loss of 0.26 kg, a BMI reduction of 0.06, a decrease in body adiposity index (−0.33), a reduction in waist circumference (−0.48 cm), and an increase in light physical activity by 32.6 min per week. However, after the devices were collected at this mark, this downward trend was no longer observed [67]. Research has shown that individuals’ psychological states primarily influence digital health engagement and outcomes [68], and we hypothesize that the findings of the Evident 3 trial may be explained by the Hawthorne effect if patients are aware that their real-time data is visible to their physician. These findings suggest the potential value of mobile health apps and wearable technology in maintaining long-term behavioral change—real time tracking and adjustments enable a constant feedback loop that could reduce the burden on providers by alerting them only when intervention is necessary, as well as fostering patient accountability. Additional insights into patient perspectives on wearable health technology and its impact on behavior and outcomes will be explored in the following section.

### 1.6. Risk Assessment

AI can play a transformative role in predicting childhood obesity risk, which affects health risks in adulthood [35]. ML has significantly accelerated this area [51,52], enabling researchers to train models on electronic health record (EHR) data [53,54] to predict future trends in obesity and identify a comprehensive range of risk factors beyond the typical diet and physical activity, including marital status, location, age group, educational background, mental–emotional disorders, and more [69,70]. One study of 327 participants, aged 21–78, with at least 9 months of self-reported follow up weight data during weight loss intervention, trained several ML models, with the best achieving prediction accuracy of >50% with half a month of data and up to 97% with 8 months [35].

AI has also been applied to publicly available health datasets to predict obesity trends through genetic, epigenetic, and environmental components [71]. For example, studies have leveraged AI to refine risk indicators like waist circumference [72] and the relationship between supermarket proximity and body weight [36].

### 1.7. Enhanced Analytics

For AI in analytics, while measuring body composition can be more diagnostic and prognostic than measuring BMI alone, manual assessment of body cross-sectional imaging via computed tomography and magnetic resonance imaging is labor intensive and time consuming [73]. By automating these processes, AI can help alleviate provider burnout while simultaneously improving accuracy and efficiency.

Furthermore, AI-driven imaging is also improving obesity care by refining cardiovascular risk stratification [74] and identifying more sophisticated biomarkers for deep phenotyping of perivascular adipose tissue in risk prediction of vascular function [75]. AI-mediated imaging can also improve outcomes for invasive procedures such as increased first-attempt procedure success rate and needle placement time for spinal anesthesia among patients with obesity [73]. These applications highlight the potential of AI to enhance both diagnostic precision and procedural success, benefiting both patients and providers.

### 1.8. Assisted Clinical Decision Making

Bariatric surgery is a common treatment for morbid obesity, however short- and long-term complication rates and weight loss remain unpredictable [76]. A systematic review about ML applications within bariatric surgery found potential in predicting postoperative complications, weight loss, decision-making, diagnosis, and postoperative quality of life. However, the lack of external validation cohorts in most studies indicates the current uncertainty surrounding these ML models [76].

In another study reviewing the role of AI in bariatric surgery, ML algorithms were shown to enhance physicians’ decision-making across several stages—from presurgical risk assessment to intraoperative management and postoperative outcomes prediction. Specifically, AI was found to improve preoperative risk assessments, identifying factors such as difficult intubation in patients with obesity, obstructive sleep apnea, pulmonary dysfunction, and hiatal hernia [77]. While the use of AI in intraoperative management is still developing, there is notable potential for AI to aid in pharmacotherapy management and hemodynamic optimization during surgery. Furthermore, AI models have demonstrated the ability to predict postoperative morbidity and mortality, including weight loss success, obesity-related disease remission, and long-term quality of life [77]. This is significant considering a survey conducted at a single institution, which assessed physicians’ understanding of bariatric surgery revealed that many providers do not initiate discussions about weight loss surgery with eligible patients. Barriers to initiating these conversations included a lack of education on patient qualifications, inefficient referral processes, and concerns over the safety of the procedure [78,79]. AI could help address these gaps by providing decision support tools, such as large language models like OpenAI’s ChatGPT-4, Microsoft Bing, and Google Bard, which can offer timely, evidence-based responses to clinical questions related to bariatric surgery [22].

Additionally, a study monitoring week-long glucose levels in an 800-person cohort developed an ML algorithm that integrated blood parameters, dietary habits, physical activity, and gut microbiota to predict personalized postprandial glycemic responses to real-life meals. This model could assist healthcare providers in offering more tailored nutrition recommendations for their patients [80].

On a system level, reinforcement learning (RL) algorithms have been used to optimize behavioral weight loss (BWL) therapies, offering a scalable, cost-effective solution for delivering personalized treatments. For patients who require lower-intensity treatments, the algorithm can identify cost-effective strategies, while for those needing more intensive interventions, it can recommend higher-cost options. This approach dynamically predicts the most effective treatment at any given point in a patient’s weight loss journey, balancing resource limitations with patient and provider satisfaction [49].

### 1.9. Potential Challenges

Healthcare provider trust and familiarity with the advent of AI technology remains an obstacle to adoption of AI technology. In one study, an ML model predicted the success of weight loss interventions with 81% accuracy at six months. To address the skepticism surrounding the model’s predictions, an explainability tool called PRIMO was developed, helping weight management experts understand and trust the model’s outputs. Interviews with healthcare providers confirmed the value of PRIMO, emphasizing that diverse explanations, uncertainty visualization, and performance metrics played key roles in bridging this gap in trust [81].

Providers need comprehensive training and education to effectively integrate AI into their practices. Additionally, concerns over AI potentially depersonalizing the patient–provider relationship persist, as does anxiety surrounding data privacy and security. Moreover, successful integration of AI into existing healthcare systems requires significant effort, as current infrastructures may not be equipped to seamlessly accommodate advanced AI technologies [82]. These barriers must be addressed to ensure AI can truly enhance obesity care without unintended negative consequences. Table 2 summarizes a list of challenges in implementing AI/ML for obesity management.

### 1.10. Patient Perspectives on AI in Obesity Care

Utilizing advanced algorithms like RL, AI optimizes interventions to serve individual patient needs, ensuring more effective outcomes with reduced resource demands. These technologies not only tailor the type and intensity of interventions in real time but also integrate diverse data sources, such as dietary habits, physical activity, and genetic and microbiome profiles, providing highly precise care.

Patients benefit from AI’s ability to deliver consistent, cost-efficient support through digital platforms including virtual health coaches, automated messaging, and gamified engagement tools which are often free to use. RL-based systems have demonstrated comparable weight reduction outcomes to traditional coaching while requiring significantly less human involvement, reducing labor costs without compromising efficacy. AI models address challenges in individual adherence and accessibility through incorporation of real-time monitoring and natural language communication, creating supportive and engaging interventions. These approaches not only improve outcomes but also optimize the patient experience through empowerment, accessibility, and sustained behavior change.

### 1.11. Real-Time and Personalized Coaching

BWL is a highly effective obesity treatment, with patients achieving reductions ranging from 7–10% of body weight [49]. Individuals are taught to self-monitor and set personal goals, while receiving consistent and frequent feedback from professional weight loss coaches. However, the scalability of BWL is limited by the shortage of expert clinicians and the high costs of delivering such labor-intensive programs. Another significant challenge in long-term follow-ups is a predominant pattern of weight regain, with fewer than 3% maintaining their post-treatment weight [92]. Studies comparing remote and in-person BWL interventions have found both to be effective, with similar average weight loss outcomes, suggesting that remote support is a viable and scalable approach to managing obesity [93].

AI methods, such as RL, are designed to enhance the efficacy and scalability of BWL by addressing its key limitations. RL-driven systems dynamically adjust the intensity and type of interventions based on real-time data, such as weight fluctuations and physical activity, to provide personalized coaching without requiring time and costs associated with extensive human involvement [49,50]. To address these challenges, a pilot study evaluated an RL-based system that dynamically adjusted intervention intensity based on participant responses through a combination of phone calls, text messages, and automated notifications [50]. In a trial involving 52 overweight or obese adults, participants were randomized to either standard weekly group treatment or AI-optimized treatment using two RL algorithms. Both groups achieved similar weight loss of approximately 7%, but the AI-based intervention required only one-third of the coaching contact [50]. Although limited statistical power precluded definitive conclusions, the findings suggest that RL-optimized systems can deliver effective weight management outcomes with significantly reduced resource demands, making them a promising solution for scalable and cost-efficient obesity care [50].

As a follow-up on the pilot study, a randomized controlled trial will assess whether a 12-month RL-based BWL program can deliver non-inferior weight loss results, while reducing costs and exploring secondary outcomes such as calorie intake, physical activity, and factors like depression, binge eating, and food addiction [49]. Current commercial health-specific AI-applications such as Noom and Omada are limited to offering behavioral suggestions. This RL approach is superior in that it adapts treatment intensity in real time rather than at fixed intervals, as seen in stepped care models that only optimize intensity at one or two pre-established points [49]. The four treatment models selected–automated message, paraprofessional-delivered call, MS-expert group, and MS-expert call–will vary in cost and intensity. It is predicted that these RL models will allow participants to receive the most suitable and cost-effective treatment while avoiding premature reduction in support for those who initially respond less effectively [49].

Advancements in AI, such as virtual assistants with natural language processing (NLP), enable more authentic, user-led communication, enhancing perceived support and improved behavioral outcomes [55]. While basic virtual assistants like “Lark” achieve weight loss comparable to in-person interventions, they are limited by pre-set questions and restricted user input [55,56]. In contrast, NLP-powered assistants offer dynamic, conversational interactions that feel more personalized and supportive, leading to better outcomes in digital behavior change interventions [57,58]. By integrating RL with digital person-to-person components, AI can address gaps in accessibility and adherence, offering scalable and personalized obesity care while overcoming barriers to traditional in-person support. Human-like digital interactions are essential to the success of these interventions, bridging the gap between technological efficiency and emotional connection.

AI-driven virtual health coaches, such as the chatbot Paola, can offer personalized guidance, improving diet, activity, and body composition [55]. A 12-week proof-of-concept study of MedLiPal, an AI-led lifestyle intervention incorporating chatbot Paola, a wearable activity monitor, a website, and a diet and activity log, demonstrated significant results in inactive adults aged 45–75 [55]. Participants increased their weekly physical activity by an average of 109.8 min, improved Mediterranean diet scores by 5.7 points, lost 1.3 kg in weight, and reduced waist circumference by 2.1 cm, with no significant changes in blood pressure [55]. These improvements in physical activity and dietary adherence exceeded or matched those achieved by prior computer-tailored interventions and were comparable to outcomes from intensive, personalized dietary counseling [37,55,59,60,61,62]. The study demonstrated excellent feasibility, with high recruitment rates, 90% retention, and no adverse events, emphasizing the scalability and potential of AI-assisted health behavior interventions [55].

Overall, AI facilitates personalized weight loss coaching by continuously adapting interventions to individual needs, optimizing resource use, and integrating diverse data sources to provide comprehensive and effective weight management solutions.

### 1.12. Precision Nutrition

AI-driven precision nutrition extends beyond analyzing dietary habits, food behaviors, and physical activity to incorporate advanced methodologies such as deep phenotyping, genomics, and microbiota profiling [38]. Deep phenotyping involves a detailed analysis of individual metabolic and phenotypic characteristics, moving beyond traditional risk factors like BMI or blood pressure to better understand disease manifestations [38]. This approach enables precise disease stratification, as demonstrated by large-scale studies like the Maastricht Study, which utilizes techniques such as dual-energy X-ray absorptiometry for body composition, cardiac electrophysiology, ocular pressure measurement, corneal confocal microscopy, and spirometry [39]. Assessment of visceral adipose tissue (VAT) accumulation, which poses a greater risk to metabolic health than subcutaneous fat, and epigenetic markers like DNA methylation in leukocytes, which may serve as surrogates for VAT biomarkers, will also be critical in assessing individual health risk [40,41,42]. Although resource-intensive, deep phenotyping offers critical insights into disease progression, paving the way for more effective, personalized interventions for obesity management [38].

Moreover, AI can enhance the personalization of weight loss programs through incorporation of genomic data, enabling precise coaching tailored to an individual’s genetic predispositions. A retrospective study explored the role of genomic data in enhancing the outcomes of a precision digital weight loss program for 393 participants over 120 days [43]. By incorporating single-nucleotide polymorphisms (SNPs) into machine learning models, the accuracy of predicting weight loss improved significantly, with mean squared errors reduced and logistic model performance enhanced [43]. Specific SNPs related to metabolic pathways, such as rs17300539_G and rs2016520_C, were linked to weight loss, with greater risk alleles associated with higher average weight loss [43]. Personalized coaching tailored to genetic risks and active participant engagement contributed to these results, with 72% of participants losing weight and 36% losing at least 5% body weight [43]. The findings suggest that integrating genomic data into digital therapeutics may improve weight loss efficacy, though further large-scale studies are necessary for validation.

AI-driven optimization of gut microbiota can play a crucial role in supporting weight loss, particularly through regulation of body weight, metabolism, and inflammation [44,45]. AI analyzes individual microbiome profiles to predict responses to dietary interventions, such as tailoring high-fiber diets for Prevotella-dominant individuals and increasing bifidobacteria for Bacteroides-dominant individuals [45]. ML models possess the ability to integrate microbiota and genetic data, allowing for personalization of diet plans and predicting weight loss outcomes based on baseline gut microbiota composition. For instance, species such as *Akkermansia muciniphila* and *Alistipes obesi* may be linked to better weight loss outcomes [46,47,94]. Beneficial species can then be targeted to optimize obesity interventions [94]. Customized dietary recommendations and probiotic formulations can then be generated by AI models to fit a person’s individual needs.

### 1.13. Sustained Lifestyle Interventions

The integration of AI into sustainable lifestyle interventions fostering long-term behavioral changes, enables the possibility of significant and sustained weight loss. AI-driven lifestyle interventions, such as SureMediks, have demonstrated significant average weight loss of 14% over 24 weeks in a multinational field trial [95]. Moreover, 99% of participants lost more than 5% of their body weight [95]. The program utilized accountability circles and gamified challenges to enhance engagement and motivation, while also tracking progress with internet-connected scales. The study found that sub-goal reassignment negatively impacted weight loss, emphasizing the importance of precise goal-setting and consistent tracking for sustained results.

AI-assisted apps like the Eating Trigger–Response Inhibition Program (eTRIP) modulate eating behaviors through features such as chatbot-based check-ins, food image recognition using computer vision, and automated nudges. These tools promote mindfulness and self-regulation, leading to significant reductions in overeating and snacking [96]. In a 12-week study testing eTRIP’s feasibility and effectiveness in a Southeast Asian cohort, participants demonstrated notable improvements not only in eating behaviors but also in depression and physical activity, with an attrition rate of just 8.4% [97]. Participants praised the app’s features, including mindful self-monitoring, personalized reminders, and its user-friendly design, indicating high satisfaction. The study demonstrates eTRIP’s potential as a scalable, personalized weight management solution, supporting its need for further evaluation on a larger scale.

Lifestyle changes adopted during the COVID-19 pandemic, including reduced physical activity and increased sedentary behavior, have contributed to rising obesity rates, particularly among adolescents [48]. The impact of the AI-based gesture recognition game Super Kids Adventure (SUKIA) and Nintendo Switch (NINS) on health metrics was evaluated in a study of 24 obese adolescents [98]. By utilizing a CNN for gesture recognition, SUKIA provides real-time visual and auditory feedback for exercises such as stretching, lunging, and squatting, which enhance back muscle strength and overall fitness [98]. Its user-friendly interface, customizable characters, and alarm function encourage regular exercise while applying deep learning for precise movement tracking. Over three weeks, SUKIA outperformed NINS in improving calorie consumption, VO2 max, and perceived exertion, demonstrating its potential as a motivational tool to enhance cardiopulmonary function and physical activity in adolescents with obesity [98].

Food addiction poses significant challenges to obesity treatment, often being linked to increased caloric intake, difficulty adhering to weight loss interventions, larger BMI, and greater weight regain after weight loss attempts [99,100]. Food addiction can also heighten internalized weight bias and body shame, reducing motivation and self-efficacy in weight loss programs, thereby emphasizing the importance of efficient tools to inform AI-driven lifestyle interventions [101]. A rapid screening tool for food addiction using ML was tested, analyzing 152 items from 176 patients to identify key predictors [102]. Using the Fast Correlation-Based Filter (FCBF), three significant items were identified: “I eat to forget my problems”, “I eat more when I’m alone”, and “I eat sweets or comfort foods”. Among nine predictive algorithms tested, the Naive Bayes classification showed the best performance, leading to the creation of a three-item nomogram based on the Yale Food Addiction Scale [102]. This simple and effective tool provides a foundation for integrating food addiction assessment into AI-powered health interventions, with future validation studies planned to confirm its clinical utility.

Sustainable lifestyle interventions can be accomplished by RL methods through personalized mobile health interventions based on timing, frequency, and type, aligning with the concept of just-in-time adaptive interventions (JITAI) [103,104]. JITAI defines momentary interventions through components like decision points and intervention options, and RL offers a practical method for tailoring these components. Unlike traditional learning methods requiring large initial datasets, RL agents learn through experience, allowing interventions to adapt dynamically [103,104]. Additionally, the learning process can be established using external expert knowledge to guide initial intervention strategies. Simulated experiments demonstrated the algorithm’s ability to tailor interventions effectively, outperforming standard RL methods and supporting personalized, just-in-time health solutions [103]. This approach shows promise for real-world applications, including the POWER2DM project, which empowers diabetic patients to self-manage their condition.

Beyond individual patient management, these AI-driven adaptive interventions have broader implications for public health efforts in obesity prevention. By leveraging real-time data and predictive analytics, public health officials can design more targeted, scalable interventions that address high-risk populations proactively. AI-powered models, such as DeepHealthNet, can predict obesity risk with high accuracy by analyzing demographic, dietary, and physical activity data, enabling better resource allocation and early intervention strategies [26]. Additionally, AI-driven meal planning systems provide personalized dietary recommendations based on caloric and macronutrient needs, supporting long-term weight management [18]. With AI’s ability to refine predictions and interventions over time, these technologies offer a powerful tool for shaping public health policies that aim to reduce obesity rates and mitigate associated chronic diseases [26].

### 1.14. Handling of Data Privacy in Weight Loss Coaching

The handling of private patient data in personalized weight loss coaching relies on methods like federated learning, hybrid techniques, data ownership, and informed consent. Federated learning enables AI models to train on decentralized devices or servers without exchanging local data, reducing the risk of breaches while enhancing privacy [84]. The decentralized approach maintains data on individual devices, eliminates centralized pooling, supports real-time learning, and benefits from diverse data sources [84,85]. Despite its advantages, challenges such as vulnerability to attacks, robustness failures, and efficiency concerns remain [85].

Hybrid methods combine federated learning with approaches like differential privacy, which adds noise to obscure individual identities, and secure multi-party computation, which allows joint data analysis without exposing private inputs [84]. These integrated techniques address privacy challenges in complex scenarios, but they face scalability issues, including high communication costs and barriers in implementing advanced methods like homomorphic encryption [88]. To overcome these limitations, research is focused on developing communication-efficient systems to make hybrid techniques suitable for large-scale healthcare applications. Additionally, data ownership and informed consent frameworks ensure patients maintain control over their data, with transparent policies that support rights like consent revocation and “the right to be forgotten” [86,87]. These combined efforts aim to balance the need for robust data privacy with the scalability required for widespread implementation of AI-driven personalized healthcare solutions.

## 2. Limitations

One significant limitation of incorporating wearable AI technology into obesity treatment is the limited ownership and use of such devices, particularly among younger patients. A national survey of children and adolescents revealed that only 30.3% were current users of wearable technology, while 51.6% had never used it in the past [90]. This limited adoption rate raises concerns about accessibility and engagement, which could reduce the effectiveness of AI-driven obesity interventions in pediatric populations.

Beyond accessibility, technical and ethical challenges also hinder AI adoption in healthcare. Data security and privacy concerns remain a major issue, as patients may be reluctant to share sensitive health data with AI systems. Patients are also more likely to prefer human medical providers [91], and resist AI-augmented care [89]. Furthermore, healthcare providers may resist AI integration without effective implementation strategies and legislative support [83]. Specifically, with regards to using AI for obesity healthcare, Black adults have the highest obesity rates in the United States at 49.9% [27], while simultaneously having lower odds of selecting an AI provider compared to other races [89]. This may indicate that social determinants impacting health utilization in low income and marginalized populations might also carry over to AI-augmented healthcare solutions [105].

The significant limitations of AI must be addressed to ensure ethical and equitable implementation. As highlighted, bias can be prevalent throughout datasets, which can lead to disparities in obesity risk prediction and management, particularly among underrepresented populations. Bias can stem from unrepresentative training data, algorithmic design, and systemic inequities embedded in healthcare systems [7,73,105]. To address these concerns, AI/ML models must undergo regular “bias audits”, be trained on diverse, high-quality datasets that account for social determinants of health, and explainable AI frameworks should be integrated to improve transparency and patient–provider trust [7,51]. Ways to do so include undergoing regular “bias audits”, debiasing word embeddings, integrating model cards into AI frameworks, and include ethicists, social scientists, and marginalized communities in AI design and deployment [106,107,108,109]. Furthermore, every additional paper that delves into these topics, such as this one, will help further build a more comprehensive database of research for AI to train on. Cultural and socioeconomic factors also influence the adoption of AI-based interventions. For instance, some countries may not give patents to interventions based in AI, individuals from lower-income backgrounds may lack access to digital health tools, and AI-driven recommendations may not align with culturally specific dietary habits [6,7,105]. Additionally, psychological factors, such as patient trust in AI recommendations or preference for personal interaction, play a role in engagement and adherence to AI-guided weight management programs [68,110]. Integrating a unique within-subject longitudinal analysis in AI-based risk analysis and treatment programs would help reduce variance based on demographics or other factors [111]. Patients’ trust in AI is often exacerbated by data privacy concerns [84,85,86,87]. With regards to this concern, extensive informed consent protocols, prior de-identification, knowledge purification programs, and federated learning models can all be utilized [85,86,87]. Moreover, equitable access to AI-driven obesity management tools should be prioritized through de-biasing and de-identifying programs, policy interventions, community-based initiatives, and user-centered design approaches that tailor recommendations to diverse populations.

## 3. Conclusions

**Figure 2:**
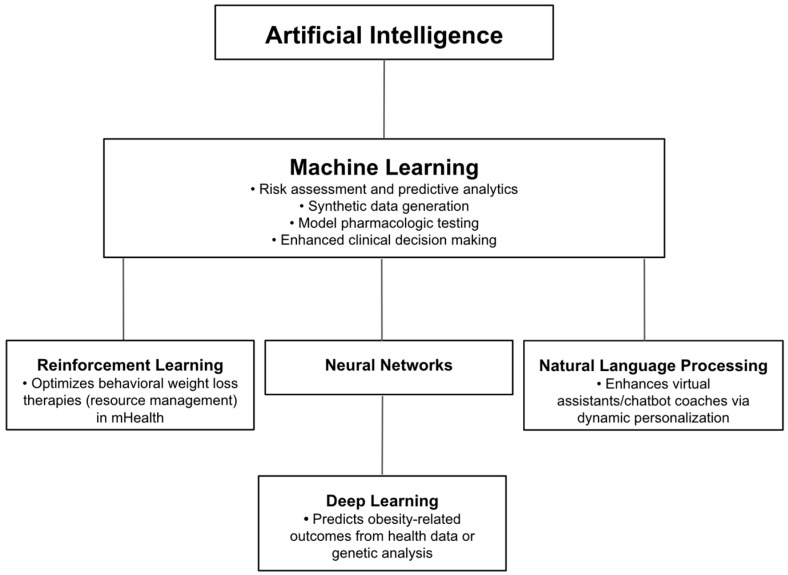
Flow chart depicting application examples of AI, ML, neural networks, deep learning, reinforcement learning, and NLP in obesity care.

The intersection of AI and healthcare presents a novel opportunity to reimagine both management and preventative strategies for childhood and adult obesity. AI-powered solutions can offer personalized interventions that can improve overall efficiency, diagnostic accuracy, and patient accessibility, a summary of techniques and application examples outlined in Figure 2.

We strongly support the integration of AI in obesity care, having explored its scalable, real-time, and sustainable potential through AI-powered coaching, precision nutrition, behavioral engagement, and preventive interventions. We recommend continued research to enhance the accuracy and effectiveness of these technologies while addressing the limitations as above. AI incorporation into obesity healthcare will require a multimodal approach that relies on thorough privacy measures, education, and cultural competency training for providers, and legislation to mitigate healthcare disparities. With continued research and collaborative efforts, AI can serve as a powerful supportive tool that empowers both patients and providers to identify personalized and cost-effective solutions for obesity and metabolic disorders.

## Figures and Tables

**Table 1:** AI Applications in childhood obesity management.

AI Application	Description	Benefits	Examples
**Risk Prediction**	Uses predictors (e.g., screen time, diet) to assess risk.	Enables early, targeted interventions. May improve resource allocation for at-risk populations in schools and communities.	Customized strategies reduce child obesity risk [29,30,31,32,35]. Social determinant mapping [36]. Genomic profiling [37,38,39,40,41,42,43,44,45,46,47].
**Gamification**	Transforms healthy habits into engaging games.	Motivates behavior change through fun methods. Builds a sense of achievement and self-efficacy among children.	“Exergames” improve fitness and weight [33,34,48].
**AI-Powered Tools**	Includes virtual coaching and active video games.	Integrates health into daily routines, providing real-time monitoring of diet, physical activity, and sleep patterns.	Apps promote activity and healthy eating [49,50].
**Family-Centered Approaches**	We suggest the involvement of families to create supportive environments.	Encourage lasting behavior changes by addressing family dynamics and preferences. Fosters collaborative goal-setting among children, parents, and caregivers.	Tailored strategies maximize impact, and family members provide consistent support. Personalized weekly messages from a clinical team to mothers resulted in higher adherence and better weight management outcomes than automated messaging [36].
**Long-Term Outcomes**	Focuses on quality of life and reduced healthcare costs.	Instills lifelong healthy habits, improving mental health outcomes and reducing the likelihood of chronic diseases.	AI-driven lifestyle changes reduce the risks of chronic diseases. Monitoring trends in AI-driven EMR analytics [51,52,53,54]. Enhanced clinical decision-making and empowered patient life coaching from chatbots [22,50,55,56,57,58,59,60,61,62].

**Table 2:** Challenges in implementing AI/ML for obesity management.

Category	Specific Challenge	Examples
**Data quality and bias**	Incomplete or biased datasets	Limited representation of diverse populations, including ethnicities and socioeconomic groups [73,83].
	Lack of explainability (“black box” problem)	Complex ML models (e.g., deep learning) are difficult to interpret [51].
	Data privacy concerns	Concerns for AI system attacks, robustness failures, and efficiency [84,85,86,87].
**Implementation barriers**	Integration into existing workflows	Difficulty incorporating AI tools into clinician practices or EHR systems. Difficulty incorporating AI into practices or EHRs due to fragmented systems, interoperability issues, and data heterogeneity [82].
	Integration into existing workflows (e.g., high cost of development and deployment)	Economic barriers for small clinics or low-resource settings [88].
	Resistance from healthcare providers	Concerns about reliance on technology and reduced human judgment [82,89]
	Equity in access to AI tools	Geographic or socioeconomic disparities in who benefits from AI-driven care [73,83].
**Patient-Centric Issues**	Limited patient trust and understanding.	Concerns over data privacy, preference for human providers, and skepticism about AI’s role in healthcare [86,87,90,91].
